# Determination of Material Requirements for 3D Gel Food Printing Using a Fused Deposition Modeling 3D Printer

**DOI:** 10.3390/foods10102272

**Published:** 2021-09-26

**Authors:** Jiwon In, Haeun Jeong, Sanghoon Song, Sea C. Min

**Affiliations:** Department of Food Science and Technology, Seoul Women’s University, 621, Hwarangro, Nowon-gu, Seoul 01797, Korea; ijw5532@gmail.com (J.I.); harnyhalo@naver.com (H.J.); sshoon@swu.ac.kr (S.S.)

**Keywords:** 3D printing, gel, gelatin, pectin, gum

## Abstract

The material requirements for printing gel food with a fused deposition modeling 3D printer were determined based on fidelity, shape retention, and extrudability, as described by the rheological parameters of storage modulus (G’), yield stress (τ_0_), and phase angle (δ). The material requirements were determined for printing gel food using three formulations containing gelatin, gelatin and pectin, and gum mixture as the gelling agents. As compared with formulations based on gelatin alone, pectin-containing gelatin-based formulations yielded higher δ and lower G’ and τ_0_ values, while gum mixture-based formulations formed a gel with higher G’ and δ values and a wider range of τ_0_. Overall, this study presents quantitative material requirements for printing gel products containing gelatin, gelatin–pectin, and gum mixtures.

## 1. Introduction

Fused deposition modeling (FDM) is the most popular three-dimensional (3D) printing method. Most of its printing parts are economical and it does not require hazardous solvents or glues [1]. Printing materials, predominantly polymers, are fed into a heating barrel in which they melt, becoming highly viscous fluids [1]. The melted materials are extruded through a nozzle and then deposited layer-wise. FDM has a wide range of applications in the fields of smart home, aerospace, and biomedicine [2]. Furthermore, FDM is currently extensively used and studied for 3D food printing [3]. The restaurant Food Ink, in the United Kingdom, recently launched a pop-up store, in which they serve full-course meals (appetizers to desserts) that were printed with an extrusion-based 3D printer. Similarly, the Italian pasta maker Barilla has been attracting attention for its unique pasta design achieved using an extrusion-based 3D printer [4]. Food manufacturing using FDM 3D printing has several advantages, including easy production of small quantities of various types of foods, allowing the consumer to prepare food anywhere and to manufacture personalized food with desired ingredients, taste, color, texture, and nutritional composition [5]. Applying 3D printing technology to gel food manufacturing enables the production of gels with exquisite shapes (which is yet to be accomplished), flavors, and aromas according to personal preferences.

However, until now, research on 3D food printing has mainly focused on the extrusion performance and visual quality evaluation of the printed products. Furthermore, there are limited studies that quantitatively correlate the physical properties of food formulations with stable printed products and adequate extrusion performance. Thus, it is necessary to determine the suitability of a formulation for printing by measuring its physical properties and comparing it with predetermined requirements. When a formulation is found to be unsuitable, the physical properties that require improving it could be identified to develop an improved formulation with the desired features. Recently, FDM printers have become increasingly popular and economical for home and restaurant use. Considering the application of research findings using these economical units, room temperature is considered the appropriate temperature for research on FDM food printing, as a temperature control system is not required to be attached to an FDM printer. Moreover, research on the requirements of the materials for 3D printing could generate various gel food recipes. Thus, the objective of this study was to propose standards for printing gels through 3D syringe-type extrusion printing with FDM using various materials based on the experimental quantification of their properties.

## 2. Materials and Methods

### 2.1. Materials

Gelatin (Jungwoodang, Seoul, Korea), high-methyl ester pectin (Genu Pec-tin type 105 Rapid Set, degree of esterification 67–73%, ES Food, Gunpo, Korea), a gum mixture, sugar (Beksul white sugar, CJ CheilJedang, Seoul, Korea), and citric acid (Citric acid S, Hwami, Incheon, Korea) were used to prepare the gel food samples. The gum mixture used in the experiment consisted of locust bean gum (50%), xanthan gum (30%), glucose (10%), agar (5%), and tamarind gum (5%).

### 2.2. Sample Preparation

The formulation of the gel food samples was determined with reference to the current scientific literature on gel products (42 in total), the composition of international gel food products in the market (23 products), and information from personal communication with a gel food product expert at Cosmax NBT (Seoul, Korea). The ingredients commonly found in gel food include sugar and acidity regulators, such as citric acid, lactic acid, and malic acid. Gelatin was the most common gelling agent (based on 18 commercial gel products and 23 publications), followed by pectin (8 commercial gel products and 12 publications). In the gelatin-based gel formulation, gelatin was in the range of 2–20% (*w*/*w*, wet basis) of the total content. In the formulations using both gelatin and pectin, gelatin and pectin were used in the ranges of 2–10% and 0.5–10% (*w*/*w*, wet basis) of the total content, respectively. Sugar, which acts as a sweetener, was used in the range of 20–80% (*w*/*w*, wet basis) to enable the formation of the gel structure and prevent syneresis after gelation [6,7], while 1% citric acid was added to the gel for acidity [8]. In addition, a gum mixture—another gelling agent—was used as a gelling agent in this study and accounted for approximately 10% of the total content. Therefore, in this study, three formulations containing gelatin, gelatin and pectin, and gum mixture as the gelling agents, with sugar, citric acid, and water were used and labeled as A, B, and C, respectively. Their compositions are listed in Table 1. The blending ratio of gelatin, pectin, gum mixture, sugar, and citric acid used in the gel production was determined by preliminary experiments. Various contents of gelatin, pectin, or gum mixture were dispersed in distilled water. Sugar and citric acid were subsequently added to the mixture and mixed. The sum of the amount of the mixed raw materials was adjusted to 100 g. For the formulation without pectin, the container was covered with aluminum foil and heated on a hot plate (85 °C) while stirring. The formulation with pectin was additionally heated for ~40 min with stirring at 85 °C using a water bath. The prepared formulation was then poured into a 30 mL Luer-Lok syringe (BD, Franklin Lakes, NJ, USA) and mounted on an FDM 3D printer.

### 2.3. 3D Printing

The printing experiment was performed with a syringe-type extrusion 3D printing system (Changxing Shiyin Technology Co. Ltd., Hangzhou, China) using the FDM method. The 3D printing system consisted of an extrusion head with a heating barrel to maintain the temperature of the formulation in the syringe, nozzle, and print platform. The extrusion head was adjusted to move along the x, y, and z axes. The 3D modeling software Autodesk 123D design (Autodesk, Inc., San Francisco, CA, USA) was used to design the 3D object (a cube with dimensions of 10 × 10 × 10 mm) to be printed. Simplify3D slicer software (Simplify3D, Cincinnati, OH, USA) was used to set the printing conditions, such as nozzle diameter, layer height, and extruder moving speed, and to slice the objects. The temperature of the extruder that comprised a heating barrel and nozzle and that of the print platform, nozzle diameter, layer height, coasting distance, and extruder moving speed used in the study are listed in Table 2. The temperature of the heating barrel, nozzle, and print platform was set at 24 °C to determine the material requirements for printing. This is because homes and small restaurants, which opt for the economical unit, can easily operate the 3D printer at 24 °C, approximately room temperature. It was difficult to immediately laminate the formulation that consisted only of gelatin, sugar, and citric acid because of the lack of a hardening property at 24 °C after extrusion from the nozzle. Thus, the movement of the nozzle was paused after stacking each layer, and the gel was covered with a cooling cup at −2 °C for 1 min to promote lamination and harden the printed layer without running down. Hence, the layer height was maintained at 0.2 mm, and the next layer was stably printed atop it.

### 2.4. Determination of the Gel Food Formulation

Three samples were printed for each formulation. The printing quality was determined based on the observation of the appearance and measurements of the dimensions of the printed sample (i.e., printability and dimensional stability). The photographs of the printed structures were immediately acquired after printing to evaluate their overall appearance, line arrangement, line shape, continuity, and filling condition [9]. The cube height was measured with a micrometer immediately and 1 h after printing. Furthermore, the printability and dimensional stability, which indicate the precision and shape stability of the printed structure, were obtained with the following equations, respectively [10].

(1) $$\mathrm{Printability}\;(\%)=\frac{\mathrm{Achieved}\;\mathrm{height}\;\mathrm{of}\;\mathrm{the}\;\mathrm{printed}\;\mathrm{object}}{\mathrm{Target}\;\mathrm{height}\;\mathrm{of}\;\mathrm{the}\;\mathrm{object}}\;\times \;100$$

(2) $$\mathrm{Dimensional}\;\mathrm{stability}\;(\%)=\frac{\mathrm{Height}\;\mathrm{of}\;\mathrm{the}\;\mathrm{printed}\;\mathrm{object}\;\mathrm{after}\;1\;h}{\mathrm{Height}\;\mathrm{of}\;\mathrm{the}\;\mathrm{printed}\;\mathrm{object}\;\mathrm{immediately}\;\mathrm{after}\;\mathrm{printing}}\;\times \;100$$

### 2.5. Determination of Printing Material Requirements

For the printing material requirements, fidelity, shape retention, and extrudability were considered. Fidelity indicates the printing accuracy of the shape and size of the printed structure compared with those of the target structure. Shape retention describes the shape stability after printing. Extrudability specifies the ease of extrusion through the nozzle [11,12,13,14]. Fidelity is characterized by the storage modulus (G’) of the material [15,16], while shape retention is characterized by the G’ and yield stress (τ_0_) of the material [16,17]. The phase angle (δ) of the material was used to describe its extrudability [18].

### 2.6. Determination of the Rheological and Mechanical Parameters

The rheological properties of the material were determined using a rotational rheometer (MCR 92, Anton Paar, Graz, Austria) with a cup-and-bob geometry (bob length, inner diameter, outer diameter, and measuring gap of 82 mm, 40 mm, 42 mm, and 0.5 mm, respectively) and were analyzed by Anton Paar Rheocompass (Anton Paar), a built-in software of the instrument. All measurements were repeated in triplicates at 24 ± 1 °C, and the average values were plotted.

G’ and δ were obtained based on the amplitude sweep tests of the rheometer [19]. To determine the linear viscoelastic region, a strain sweep was conducted in the oscillation test mode in the range of 0.1–100% at a fixed frequency of 1 Hz during a test time of 200 s [12]. G’ and δ were calculated as the average values in the linear viscoelastic region [9,17,20,21]. The yield point was determined as the initial drop section of G’, i.e., the point at which, upon increasing the applied stress, the solid first shows liquid-like behavior [22]. The starting section of the nonlinear region appeared because of the structural breakdown of the sample; the stress at this time was considered as τ_0_ [13].

## 3. Results and Discussion

### 3.1. Determination of the Gel Food Formulation

The observation results and quality measurements of the printed gel foods with various compositions are listed in Table 3. As the gelatin content increased, the printed 3D structure became more stable under the same printing conditions. However, at gelatin concentrations of ≥20%, the formulations became sticky with increased viscosity. Consequently, there were inconsistent extrusion patterns with drop-shaped lumps that formed at the tip of the nozzle during printing, indicating their unsuitability for additive manufacturing. In contrast, formulations with <12% gelatin demonstrated a stable extrusion without clumping or clogging the nozzle. However, it was difficult to obtain a cube shape because the previous layers were not hard enough, despite using a cooling cup. This resulted in the spreading of the shape within the bulging center. Formulations with a gelatin concentration in the range of 14–18% (A1, A3, and A5) had uneven surfaces, resulting in unstable layered structures. Although these formulations printed and maintained cubic shapes, deformations were observed on the top of the printed samples (Table 3). When the sugar content was varied while fixing the gelatin content at 14, 16, or 18%, the viscosity of the formulations containing 50% or more sugar increased. This resulted in the materials stretching like a thread at the nozzle, which was not suitable for extrusion printing. Meanwhile, the formulations with 40% sugar (A2, A4, and A6) exhibited excellent shape stability even with internal filling owing to the seamless and stable extrusion of the materials through the nozzle along a line with consistent thickness.

Based on the appearance of the printed samples at different gelatin concentrations with fixed pectin (7%), sugar (30%), and citric acid (1%) contents in the B formulations, formulations with ≥12% gelatin were unsuitable for printing owing to their high viscosity, whereas those with 10% gelatin were easily extruded, forming the intended cubic shape. Furthermore, although the formulation with 8% gelatin demonstrated excellent adhesion between layers, it was difficult to print an accurate cube shape, as the printed sample was easily disturbed by the moving nozzle during printing because of insufficient hardening. In addition, the printed sample was unable to support its own weight and began spreading. As the pectin content increased with the gelatin concentration fixed at 10%, more solid and stiff formations were obtained.

Regarding the effect of the gum mixture concentration on the appearance of the printed samples with fixed 30% sugar and 1% citric acid contents, the formulation with 8% gum mixture (C1) formed a thin and weak gel, which was not hard enough at room temperature (24 ± 2 °C) and could not withstand its own weight. In contrast, a cube shape was printed with the formulations containing 10–12% gum mixture (C3 and C5); however, the line extruded through the nozzle was cluttered, making it difficult to deposit a flat layer. When the concentration of the gum mixture was fixed at 8, 10, or 12% with increased sugar concentration, printed samples with high resolution and uniform and smooth surfaces were obtained. As the sugar concentration increased, the viscosity of the formulation increased, allowing a seamless and stable extrusion without broken extrudate threads [10].

A2, A4, A6, B4, B5, B6, C2, C4, and C6 formulations had adequate printing characteristics based on visual inspection; the layers were substantially fused. The formulations demonstrated printability in the range of 89.03–97.68% and dimensional stability in the range of 94.32–98.86%. These values denote appropriate printing precision and shape stability that are suitable for additive manufacturing with 3D printing. Previously, 3D printed samples with outstanding printing precision formed with various gums mixed with orange concentrate wheat starch blends have exhibited printability and dimensional stability in the ranges of 83.53–99.39% and 81.54–99.39%, respectively [10]. Therefore, the physical properties of the gel formations, which were found to be suitable for printing, were analyzed to determine the material requirements for 3D gel food printing.

### 3.2. Determination of Printing Material Requirements

#### 3.2.1. Fidelity

The rheological parameters that represent fidelity for the gelatin, pectin, and gum mixture formulations are listed in Table 4. Compared with the target dimension, the shapes and sizes of the printed samples were highly accurate (i.e., high fidelity), with excellent printability based on visual observation.

As the concentrations of gelatin, pectin, and gum mixtures in the A, B, and C formulations increased, the G’ values increased from 3539.70 to 7597.08, 208.74 to 470.48, and 6577.77 to 14,287.78 Pa, respectively (Table 4). The gelation occurred during the cooling of the gelatin dissolved in an irregular coil from the heating solution. Upon cooling, small regions, made of polypeptide chains that tend to return to the triple helical structure, were cross-linked to form a 3D network [23]. Meanwhile, as the concentration of gelatin increased, a denser gelatin network was formed, improving the ability of the gel to entrap water. Therefore, the increase in G’ due to the increased gelatin concentration was attributed to the formation of a dense network.

As the concentration of pectin increased, the strength of the pectin gel network increased, forming gels with increased elasticity and firmness [24]. Furthermore, this phenomenon increased the G’ values, which indicate the firmness/consistency of the gel structure [24]. Notably, gums have been used as gelling agents for formulations to improve the cohesiveness, elasticity, and adhesion of the gel [25]. As the gum content increased, gelation was promoted, and the tissue of the gel was hardened, resulting in an increase in G’.

The tangent of the δ (tan δ) values for A2, A4, A6, B4, B5, B6, C2, C4, and C6 formulations were in the range of 0.08–0.67. A gel with a solid-like structure, low fluidity, and prevalent elastic behavior can be formed when tan δ < 1, whereas one with prevalent viscous behavior is formed when tan δ > 1 [26]. In this study, formulations that yielded excellent printing precision formed a fairly elastic gel network. As the gelation concentration increased from 14% to 18% in the A formulation, tan δ decreased from 0.10 to 0.08, indicating that the formulation had relatively more solid-like rheological properties with poor fluidity. This may be related to the frequent thread breakage during printing and a sticky formulation due to its high viscosity, thereby resulting in a disrupted extrusion through the nozzle when the gelatin concentration exceeded 20%. The decrease in tan δ from 0.67 to 0.55 with increasing pectin concentration from 7% to 9% in the B formulations was attributed to the formation of a firmer pectin gel structure with better elastic properties [24]. Based on these results, the printing requirements of the formulations that resulted in highly accurate shapes and sizes of the printed samples with respect to the target dimension are summarized in Table 4. Therefore, a food item with the desired shape and size can be produced with excellent fidelity using a 3D printer by adjusting the physical properties of the printed materials to meet the requirements.

#### 3.2.2. Shape Retention

The analysis of the rheological parameters of A2, A4, A6, B4, B5, B6, C2, C4, and C6 formulations, which previously exhibited shape stability upon visual inspection and high values of dimensional stability (≥81.5%, i.e., high shape retention) [10], demonstrated that the G’ values of the formulation with gelatin, pectin, and gum mixtures were in the range of 3539.70–7597.08, 208.74–470.48, and 6577.77–14,287.78 Pa, respectively (Table 4). The G’ value reflects the mechanical strength (structural strength) of the material [18]. A formulation with sufficient mechanical strength (G’) can endure its own weight over time after printing, maintain the printed shape, and have excellent resolution [18]. This is demonstrated by A6 (a gelatin-based) and C4 and C6 formulations (gum mixture-based), which had high G’ values and maintained stable structures after printing with high-dimensional stability.

**Figure 1:**
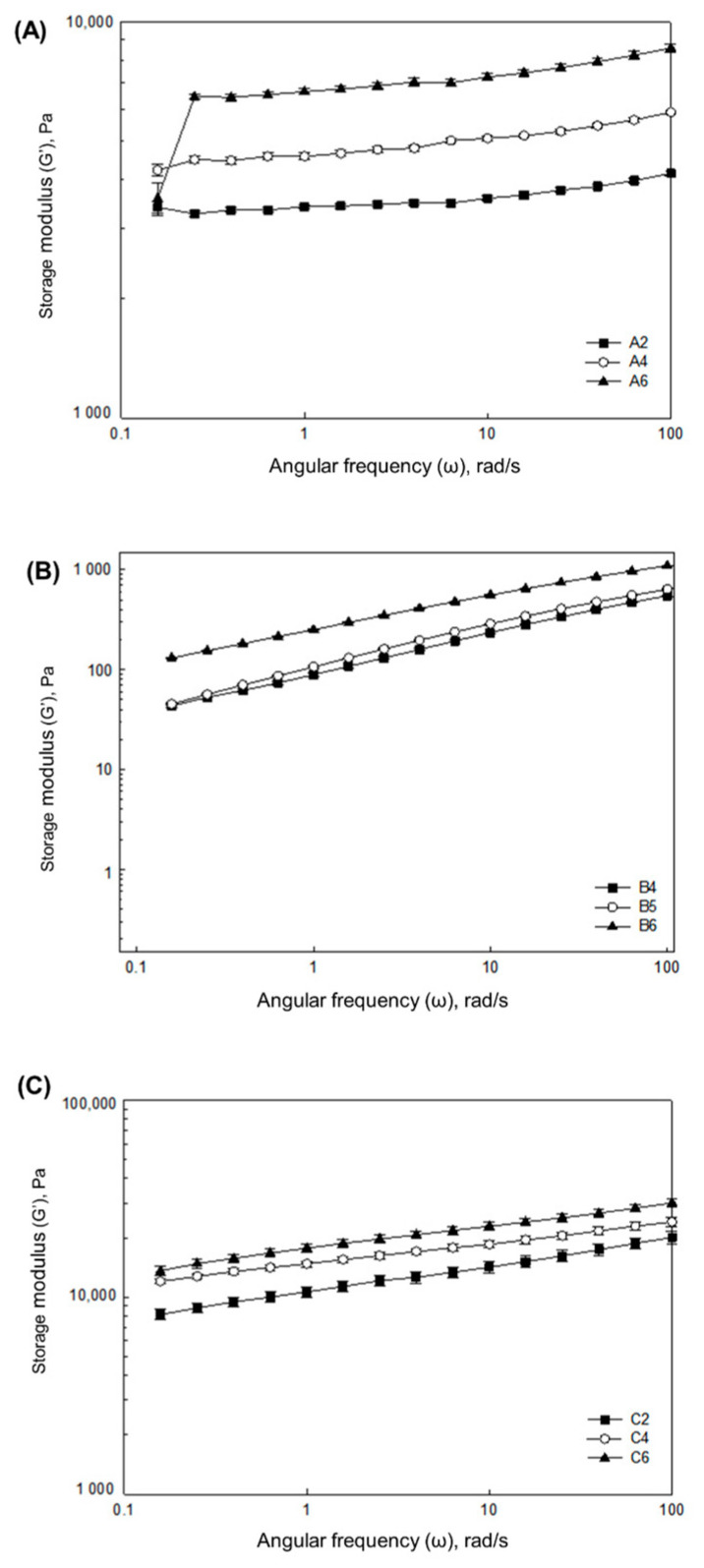
Oscillatory frequency sweeps for the selected gel formulations of A (**A**), B (**B**), and C (**C**) for 3D printing. A2, A4, and A6; B4, B5, and B6; and C2, C4, and C6 are the selected formulations from the formulations of A, B, and C, respectively.

The variations in the G’ values as a function of the oscillatory frequencies of the A, B, and C formulations with different amounts of gelatin, pectin, and gum mixtures are shown in Figure 1. Small changes in the viscoelastic properties demonstrated by the small changes in the modulus values (with respect to the frequency) in formulations A, B, and C suggested the formation of a strong and self-supporting gel with high shape retention [11,27]. In formulations A, B, and C, as the gelatin, pectin, and gum mixture concentrations increased, the G’ value increased, implying that a higher mechanical strength was achieved (Table 4). This can be attributed to the formation of a denser network structure, as more gelatin molecules absorbed water and swelled with a higher gelatin concentration.

As the concentration of pectin increased from 7% to 8% and 9% in the B formulations, the τ_0_ value increased from 207.06 to 224.41 and 281.45 Pa, respectively. High-methoxyl pectin forms gel when the non-covalent bonding of the adjacent pectin chains is interconnected to form a 3D network. The increase in the G’ and τ_0_ values at increased pectin concentrations can be attributed to the increase in the number of elastically active chains owing to the increase in the number of junction zones [24].

The main ingredients that comprise 80% of the gum mixture used in this study were locust bean and xanthan gums. The two gum mixtures, when used together, demonstrated a synergistic effect in forming a solid and thermoreversible gel with improved syneresis and enhanced stability compared with their individual use [28]. This explains the excellent shape stability of the C formulations.

The τ_0_ values related to the shape retention of the printed objects were in the ranges of 258.30–283.09, 207.06–281.45, and 204.21–295.15 Pa for formulations A, B, and C, respectively. The τ_0_ value of the A6 formulation, which demonstrated the highest dimensional stability (98.60%) among the A formulations, was higher than the τ_0_ value of A2 formulation (dimensional stability: 94.32%), which slightly collapsed after printing. For the B and C formulations with pectin or gum mixtures, the τ_0_ values of the formulations with higher dimensional stability (B6 and C6) were higher than the corresponding values of the formulations with lower dimensional stability (B4 and C2). Therefore, formulations with higher G’ and τ_0_ values resulted in outstanding dimension retention after extrusion based on the previous finding, wherein an increase in G’ with respect to an increase in dimensional stability was observed. The τ_0_ and mechanical strength were related to their ability to maintain the shape of the printed sample without collapse owing to the gravity applied to the material and stress generated by the material layer deposited thereon. The material requirements for printing the 3D structure with the gelatin, pectin, and gum mixture formulations that can support their own weight could be determined from the results (Table 4). Therefore, formulations that meet the requirements for a 3D printed sample with excellent shape retention could produce food products that retain their shape over time.

#### 3.2.3. Extrudability

The δ, related to the extrudability, is presented in Table 4. From the analysis of the rheological parameters of formulations A, B, and C that exhibited excellent extrudability through the nozzles during printing, the values for δ ranged from 4.78° to 5.88°, 28.79° to 33.96°, and 7.55° to 12.86°, respectively. For the viscoelastic materials, the δ was in the range of 0–90°. As the elasticity of the formulation increases, the δ approaches 0°. Meanwhile, as the viscosity increases, it approaches 90° [27]. For gels, the typical δ was in the range of 1.2–64°, in which elasticity-dominant gel-like structures have a δ smaller than 45° [11]. When the δ was smaller than 10°, the formulation behaved similarly to a viscoelastic solid [11]. Therefore, a solid-like gel structure with high elasticity was formed as the gelatin and gum mixture contents increased, which decreased the δ. In general, formulations with a large δ (45–90°) are liquid and non-self-supporting, whereas those with a small δ (0–3°) cannot be easily extruded through narrow nozzles [11]. In this study, A2, A4, and A6 formulations (δ: 4.78–5.88°) and C2, C4, and C6 formulations (δ: 7.55–12.86°) were stably extruded without breaking during printing and exhibited excellent dimensional stability in the range of 94.32–98.86% (Table 3). A previous study reported a formulation composed of agar, carrageenan, gellan, and xanthan-gelatin with a δ in the range of 3–15°, resulting in self-supporting hydrocolloids with good extrudability [11], which is similar to the δ of the formulation in this study. Conversely, B4, B5, and B6 formulations containing pectin had a δ value of 28.79–33.96° that suggested lower elastic properties than those of the A and C formulations, which was reflected in their relatively high δ values (Table 4) [27]. Therefore, despite their high-printing accuracies (printability: 96.79–97.68%), the dimensional stability of the B formulations was relatively lower compared with the A and C formulations. These results are in line with the results of Gholamipour-Shirazi et al. [11], in which the δ of their resulting formulation was in the range of 15–45° when non-self-supporting semisolids were printed with guar, locust bean, and xanthan gums.

Therefore, the material requirement, with respect to the δ, for seamless FDM-type gel printing (no nozzle clogging) that could achieve suitable extrudability could be determined (Table 4). Seamless printing with formulations in compliance with the material requirements would enable food production with uniform surfaces through FDM-type printing.

### 3.3. Effects of the Use of Pectin and the Gum Mixture on the Printing Material Requirements

The addition of pectin to the gelatin-based formulation (B formulation) increased the values of δ and decreased the values of G’ and τ_0_, compared with the gelatin-based formulation (A formulation). The conspicuous increase in δ and decrease in G’ values indicate that the mixing of gelatin and pectin could form a less elastic gel than that without pectin.

The G’ and δ values for the gum mixture-based formulations were higher, and the range of the τ_0_ value was wider than those of the gelatin-based formulation. Compared with the gelatin-based formulations, the gelation of the gum mixture-based formulations resulted in a stronger and more elastic gel, which may be owing to the synergistic effect of the locust bean gum and xanthan gum on the formation of a solid gel [29].

## 4. Conclusions

This study determined the material requirements for fidelity, shape retention, and extrudability that were suitable for printing gel food made of gelatin, pectin, and gum mixtures using a FDM 3D printer. Furthermore, the results suggest that the mixing of gelatin and pectin could form a less elastic gel than one without pectin. Further, the gel formed with the gum mixture yielded a stronger and more elastic gel compared with the gelatin-based formulations. The results of this study are expected to contribute to the commercialization of 3D printing technology in manufacturing gel food using various materials by tailoring the physical properties of each formulation to meet the imposed requirements.

## Figures and Tables

**Table 1:** Formulations of the samples investigated in this study.

Sample	Gelatin (g)	Pectin (g)	Gum Mixture (g)	Water (g)	Sugar (g)	Citric Acid (g)
A1	14	0	0	55	30	1
A2	14	0	0	45	40	1
A3	16	0	0	53	30	1
A4	16	0	0	43	40	1
A5	18	0	0	51	30	1
A6	18	0	0	41	40	1
B1	8	7	0	54	30	1
B2	8	8	0	53	30	1
B3	8	9	0	52	30	1
B4	10	7	0	52	30	1
B5	10	8	0	51	30	1
B6	10	9	0	50	30	1
C1	0	0	8	61	30	1
C2	0	0	8	51	40	1
C3	0	0	10	59	30	1
C4	0	0	10	49	40	1
C5	0	0	12	57	30	1
C6	0	0	12	47	40	1

**Table 2:** Three-dimensional (3D) printing parameters used in this study.

Processing Parameter	Value
Heating barrel temperature	24 °C
Nozzle temperature	24 °C
Print platform temperature	24 °C
Nozzle diameter	0.60 mm (0.84 mm for printing gum-mixture-based formulations)
Layer height	0.2 mm
Coasting distance	4 mm
Extruder moving speed	6 mm/s

**Table 3:** Printing height and moldability of the printed gel as a function of gelatin, pectin, gum mixture, sugar, and citric acid content.

Sample	Printed Products	Printing Height (mm)	Printing Height after 1 h (mm)	Printability (%)	Dimensional Stability (%)	Observations
A1	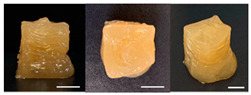	8.25 ± 0.2	7.10 ± 0.2	82.54	86.06	Uneven layer on the top of the printed object
A2	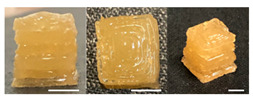	9.00 ± 0.0	8.49 ± 0.1	90.09	94.32	Good molding
A3	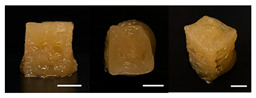	8.40 ± 0.1	7.35 ± 0.2	83.99	87.55	Uneven layer on the top of the printed object
A4	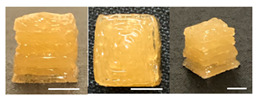	9.65 ± 0.1	9.36 ± 0.2	96.49	96.96	Good molding
A5	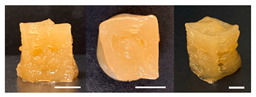	8.40 ± 0.1	7.45 ± 0.1	83.90	88.78	Uneven layer surface and unstable internal filling
A6	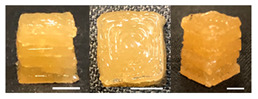	8.90 ± 0.2	8.78 ± 0.1	89.03	98.60	Good molding but there are broken threads
B1	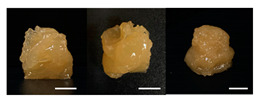	7.84 ± 0.2	5.55 ± 0.1	78.41	85.01	No basic form and spreads after printing
B2	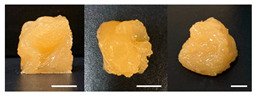	8.05 ± 0.1	6.67 ± 0.1	80.45	75.23	No basic form and spreads after printing
B3	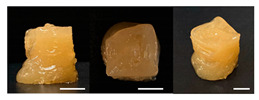	9.01 ± 0.2	6.38 ± 0.1	90.12	70.84	Good adhesion between layers but not enough hardening
B4	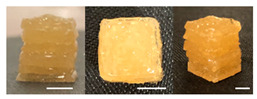	9.77 ± 0.1	9.21 ± 0.1	97.68	94.32	Good molding
B5	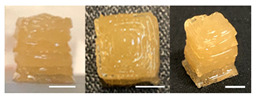	9.71 ± 0.2	9.27 ± 0.1	97.05	95.48	Good molding
B6	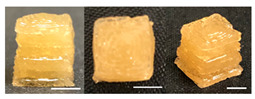	9.68 ± 0.1	9.38 ± 0.1	96.79	96.96	Good molding
C1	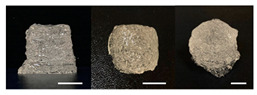	8.88 ± 0.2	8.40 ± 0.2	88.76	94.66	Weak gel formulation, poor shape retention
C2	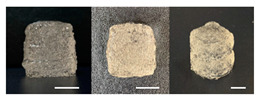	9.53 ± 0.1	9.29 ± 0.3	95.32	97.45	Good molding
C3	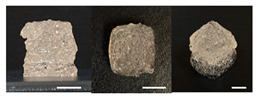	9.00 ± 0.1	8.47 ± 0.2	89.99	94.11	Cluttered line and uneven layer
C4	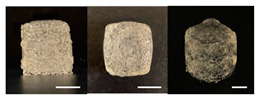	9.51 ± 01	9.32 ± 0.2	95.06	98.06	Good molding
C5	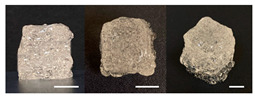	9.08 ± 0.1	8.65 ± 0.1	90.78	95.34	Cluttered line and uneven layer
C6	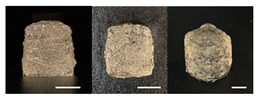	9.49 ± 0.1	9.68 ± 0.1	94.89	98.86	Good molding

For the formulation consisting of gelatin, water, sugar, and citric acid, a cooling cup (−5 °C, 1 min) was used for three-dimensional (3D) printing. All scale bars are equivalent to 5 mm.

**Table 4:** Rheological parameters for the fidelity, shape retention, and extrudability of the gel formulations as a function of the gelatin, pectin, gum mixture, sugar, and citric acid contents.

Sample	G’ (Pa)	τ_0_ (Pa)	δ (°)
A2	3539.70 ± 18.80	258.30	5.88 ± 3.56
A4	5410.13 ± 88.41	267.28	5.04 ± 0.21
A6	7597.08 ± 170.43	283.09	4.78 ± 1.80
B4	208.74 ± 7.78	207.06	33.96 ± 0.77
B5	247.95 ± 12.34	224.41	33.64 ± 0.97
B6	470.48 ± 26.22	281.45	28.79 ± 0.99
C2	6577.77 ± 115.53	204.21	12.86 ± 0.13
C4	8754.99 ± 50.71	280.32	9.81 ± 0.07
C6	14,287.78 ± 91.24	295.15	7.55 ± 0.02

## Data Availability

The data presented in this study are available on request from the corresponding authors.
